# Creation of a Tibia Extension Model With a Perforated Ilizarov Ring System: An Experimental Study in Rats

**DOI:** 10.7759/cureus.75545

**Published:** 2024-12-11

**Authors:** Burçin Karslı, Ahmet Mesut Kayacan, Nevzat Gönder

**Affiliations:** 1 Department of Orthopaedics and Traumatology, Gaziantep University Faculty of Medicine, Gaziantep, TUR; 2 Department of Orthopaedics and Traumatology, Republic of Turkey Ministry of Health Şırnak State Hospital, Şırnak, TUR

**Keywords:** experimental, extension model, ilizarov, osteotomy, tibia

## Abstract

Distraction osteogenesis is a valuable clinical technique used to address length discrepancies in long bone deformities. This procedure involves performing an osteotomy at an appropriate site in the bone and correcting the deformity through an extension system. This research aims to investigate the efficacy of a newly developed device for use in rat tibias and to provide an alternative to existing devices used in animal experiments.

A total of 16 male Wistar-Albino rats, each weighing approximately 300-350 grams and aged 20-28 weeks, were used in the study. On the first day, a fixator was applied to the right tibias of the rats, and Ilizarov osteotomy was performed. Distraction of the tibia commenced on the Day 7. During the distraction phase, which lasted seven days, tibia lengthening was performed twice daily at 08:00 and 16:00, with each session involving a distraction of 0.25 mm. After a 14-day waiting period, evaluations were conducted on the 28th, 35th, 42nd, and 49th days post-surgery.

Following these assessments, the rats were evaluated with X-rays and subsequently sacrificed. The distraction procedures proceeded largely without issues in the rats. Union was observed during the follow-up after distraction. In the initial postoperative X-ray of one rat, no problems with the reduction of the osteotomy were detected. To verify the functionality of the system, acute distraction was tested in one rat model, and successful elongation was achieved. However, one rat experienced circulatory disturbances post-operation, with the extremity showing an ecchymotic appearance. The extremity returned to its normal state during follow-up. Infection occurred in three rats. No postoperative antibiotic therapy was administered to any of the rat models.

During follow-up, the infections resolved with regular dressing changes. Due to fewer complications and improved radiological imaging with the extension models performed in the metaphyseal region of rat tibias, our system could be utilized in future fracture model applications or distraction osteogenesis studies involving rat models. We believe it could serve as an alternative to other models for creating extension models at a lower cost.

## Introduction

Distraction osteogenesis is a valuable clinical technique used to correct deficiencies in long bone deformities, congenital diseases, trauma, and skeletal deformities and defects caused by tumors [1-4]. This procedure involves performing an osteotomy at the appropriate location on the bone and addressing the deficiency using an elongation system. During the postoperative period, patients may face issues such as prolonged fracture healing, pin site infections, and angulation.

Distraction is defined as the pulling of an extremity in two different directions. Distraction osteogenesis refers to the formation of new bone between two separated vascularized bone surfaces. If the distraction process is conducted under appropriate conditions, it occurs through intramembranous ossification. Major factors affecting healing include the patient's age, the site of osteotomy, the distraction interval, the amount of soft tissue, and the surgical approach [1]. In a stable distraction, the bone ossifies intramembranously, while in unstable distractions, it heals through endochondral ossification. If there is excessive instability, pseudoarthrosis may develop [2]. Distraction osteogenesis has evolved over time with the development of skeletal traction, bone segment fixation, and osteotomy techniques. Distraction osteogenesis was first applied by Codivilla, and years later, Ilizarov expanded its use in orthopedic surgery by elucidating the physiological mechanisms of associated bone regeneration [3-4]. 

Today, distraction osteogenesis is widely used in the treatment of segmental loss and deformities in bones resulting from trauma, infection, or tumor resection [1-4]. Distraction osteogenesis consists of three stages: the latent phase, the distraction phase, and the consolidation phase [5]. The stages of distraction osteogenesis progress similarly in both humans and rats.

Latent stage

The period between the initiation of traction following an osteotomy created in the bone and the formation of callus is referred to as the latent phase. This period histologically resembles fracture healing [6]. Initially, a hematoma forms at the osteotomy site. Over time, the hematoma evolves into a clot, followed by necrosis of the fractured bone. Five days after the osteotomy, the distal and proximal parts of the osteotomy site begin to form capillary loops within the medullary canal [7]. The newly formed network system at the capillary terminal region contains mesenchymal and free stem cells. This initiates the synthesis of new bone.

Distraction stage

The distraction phase is the stage during which traction is applied. In this phase, new bone formation and distraction regeneration occur. During elongation, the normal fracture healing process is disrupted by the traction. Soft callus creates a new microenvironment due to the tension stresses between the fragments. This microenvironment progresses in parallel with the tissue formation throughout the traction [8,9]. The spindle-shaped cells localized to the collagen fibers are arranged in parallel with the collagen fibers. These spindle-shaped cells within the collagen fibers spread to both ends of the distraction area, dividing into two groups. During Days 7-9 of distraction, there is an increase in capillaries towards the fibrous tissues. This condition allows vascular expansion to extend into both the bone cavity and the medullary canal. The formed capillary networks are aligned parallel to the elongation area [7,10-12]. The vascularization in the elongation area shows a tenfold increase compared to the normal fracture site. As the void in the elongation area increases, the bone columns elongate and thicken. The formation of new structures in the elongation area continues as long as the distraction persists [5].

Consolidation stage

This period is defined as the time required for the maturation and corticalization of the osteotomy line after the cessation of distraction. It is twice the duration of the distraction period. After the completion of elongation, the fibrous gap zone gradually ossifies, forming a woven bone zone. Initially, the distraction area forms as intramembranous ossification. Over time, zones related to primary osteons are resorbed and disappear. In subsequent periods, the initially formed parallel fibrin transforms into lamellar bone. This strengthens bone development. Both cortical and medullary bones are eventually repaired. The cortical bone structure is remodeled to its original state through Haversian systems, with this process taking approximately one year or more [12,13].

The aim of this study is to assess whether our newly developed ring system works in rats by performing osteotomies and to provide an alternative to elongation models. We aim to clinically and radiologically evaluate the ring system on rats. Our perforated Ilizarov system could serve as an alternative to other models for creating extension models at a lower cost.

## Materials and methods

In the study, 16 Wistar-Albino male rats, each weighing an average of 300-350 grams and aged 20-28 weeks, were used. The study was conducted with the approval of the Gaziantep University Experimental Research and Development Center Animal Experiments Local Ethics Committee, under approval number 2023/43. Throughout the experiment, the rats were housed individually in separate cages. Pellet rat chow was used before and after surgery, and water was provided ad libitum and changed daily. All animals were maintained and fed under conditions of 12 hours of light and 12 hours of darkness at 24°C room temperature.

Study design

For our study, a total of 16 rats were used to create elongation models, with four groups of four rats each. The groups were categorized based on the waiting periods after the elongations were completed. After the elongations were finished, the animals in the groups were sacrificed following waiting periods of two, three, four, and five weeks. Imaging and complications in the elongation areas were assessed before sacrifice. The elongation areas were evaluated using imaging techniques.

Fixator setup

**Figure 1:**
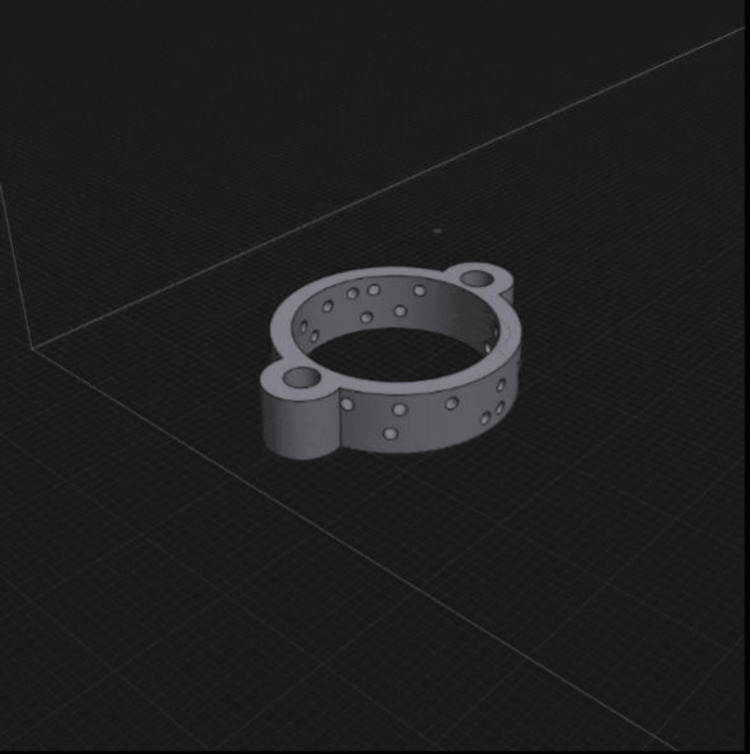
The designed ring in the Shapr3D™ (Budapest, Hungary) program.

The ring systems used for distraction osteogenesis were designed using the free educational version of the 3D design program Shapr3D™ (Shapr3D Zrt., Budapest, Hungary) (Figure 1). A ring system was designed based on the average diameter of the femur circumference measured in rats. The design included holes to accommodate K-wires at various angles. To connect the two rings, 3mm diameter holes were added to the sides of the rings, allowing them to be joined with two 3mm diameter screws and nuts. The elongation process was planned to be achieved through these nuts. The designed rings were printed using polylactic Acid (PLA) filaments with the Ender-3 V2 printer from Creality™ (Shenzhen Creality 3D Technology Co. Ltd., Shenzhen, China). PLA is a plastic produced from products like corn starch, sugarcane, and sugar beet. Since PLA is made from entirely organic materials, it is a biodegradable filament and poses no adverse health effects. It is also cost-effective and readily available.

Surgical procedure

**Figure 2:**
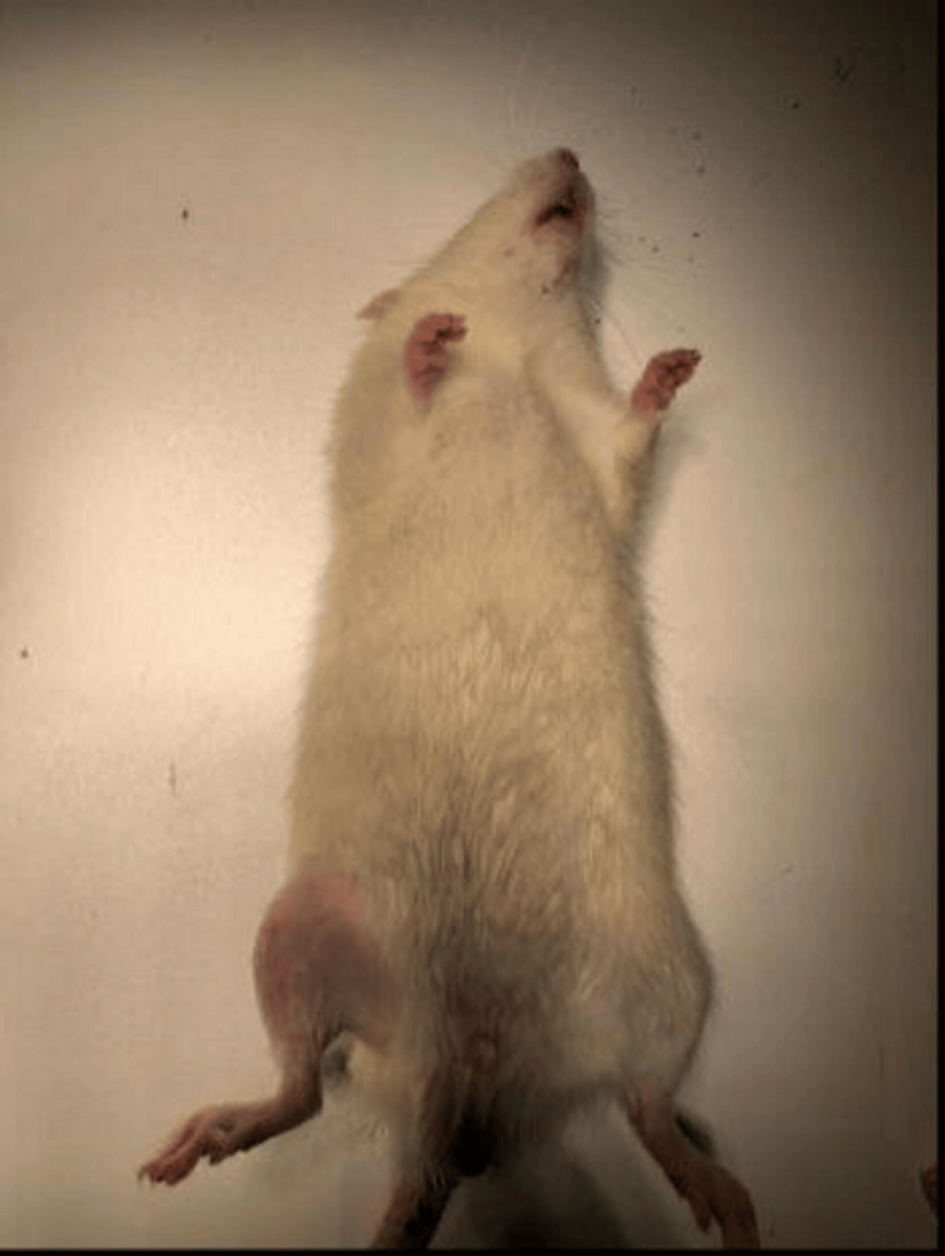
Surgical Preparation of the Rat Model Ready for Operation

The procedure was done under general anesthesia. Ketamine HCL 100 mg/kg was given intraperitoneally as a single dose. Additionally, xylazine HCL was given as a single dose of 10 mg/kg intraperitoneally. Meloxicam was administered as analgesia at a dose of 1-2 mg/kg once a day. After general anesthesia was administered to the rats, they were placed in a supine position, and the hair on the right hind limb was shaved (Figure 2).

**Figure 3:**
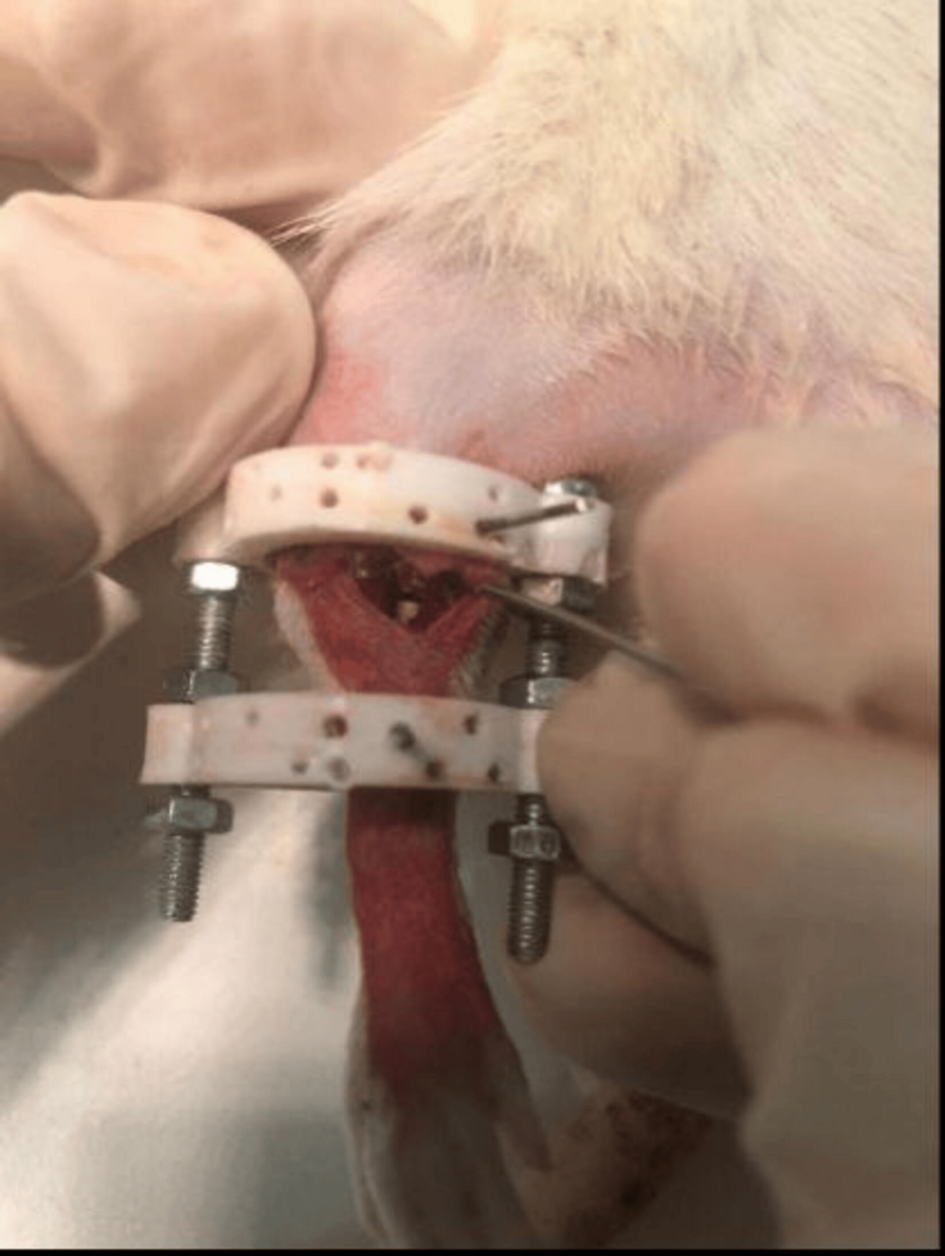
Rat Model With Tibial Osteotomy and Fixator System Installed

An aseptic surgical field was prepared, and the lower extremity was painted with betadine solution while maintaining sterilization conditions throughout the procedure. To apply the fixator at the correct level, the malleolus was palpated, and a K-wire was inserted from the proximal to the medial side laterally. Then, another K-wire was inserted in the same manner just distal to the knee to establish the fixator system. The skin and subcutaneous tissues were incised to reach the tibia for osteotomy. Once the tibia was reached, an osteotomy was performed using the Ilizarov technique with the aid of a drill [14] (Figure 3). Osteotomy was performed in the proximal metaphyseal region in all rats. The fibula was manually fractured in all rats [15]. Seven days later, lengthening of the tibia was initiated using the fixator system. Daily lengthening of 0.25 mm was performed twice a day, continuing for a total of seven days. Following this period, callus formation was anticipated. The rats were then observed with waiting periods of 14, 21, 28, and 35 days, after which they were sacrificed.

Lengthening technique

On the first day, a fixator was placed on the right tibia of 16 rats, and an Ilizarov osteotomy was performed [16]. On the seventh day, tibial lengthening began. For seven days, lengthening was performed twice daily at 08:00 and 16:00, with each extension set to 0.25 mm [17]. Following a 14-day interim period, evaluations were performed on the 28th, 35th, 42nd, and 49th days following the surgery. In the imaging of the rats, radiographs were taken using Hasvet 838R (plx102) veterinary mobile X-ray equipment (Hasvet Medical Co. Ltd., Antalya, Türkiye) at the veterinary clinic and the amount of lengthening was measured and calculated from the X-ray images. Then the rats were sacrificed by cervical dislocation under anesthesia.

In order to find the expectation of a very large effect size (d=2.62) among the physiological parameters of the study group to be statistically significant, the minimum number required in each group was determined as 4 (α=0.05; 1-β=0.80). In order to keep the experimental and control groups balanced, it was decided to include the same number of subjects in each group. Power analysis was performed in G*power 3.9.1 software (Heinrich-Heine-Universität Düsseldorf, Düsseldorf, Germany).

Postoperative care

After the surgical procedure, no group of rats was left in immobilization. It was intended to be mobilized by pressure following the surgical procedure. The rats were confined to metal cages during the postoperative period. The rats in these enclosures were nourished with water and standard rat chow (ad libitum) and were monitored under fluorescent light and a constant room temperature (24° C). The surgical area was dressed daily with betadine in the postoperative period.

## Results

In our study, a total of 16 animals were used, with four rats in each of the four different groups. Each group was monitored for two, three, four, and five weeks after the lengthening procedures were completed. After the designated period, X-rays of the right tibias of the rats were taken, and the animals were then sacrificed for examination. One animal was excluded from the study due to death, so all procedures were evaluated based on 15 animals. The rats were assessed within their groups, and developments were recorded.

**Figure 4:**
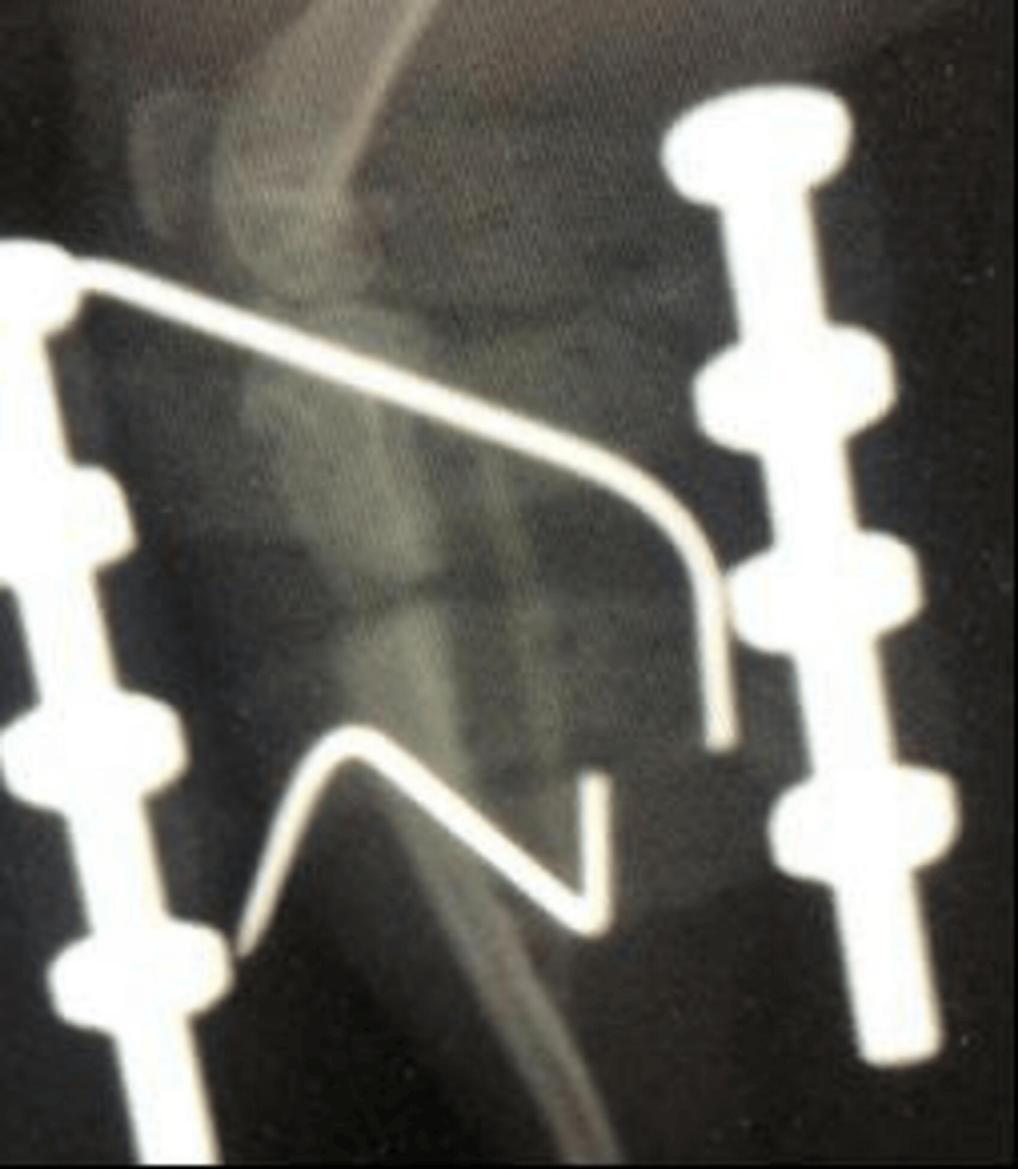
First Post-Operative X-Ray of the Rat

Most of the rats underwent the lengthening procedures without major issues. Follow-up after the lengthening showed that union was achieved. After the osteotomy was performed, the initial post-operative X-ray showed no problems with reduction (Figure 4).

**Figure 5:**
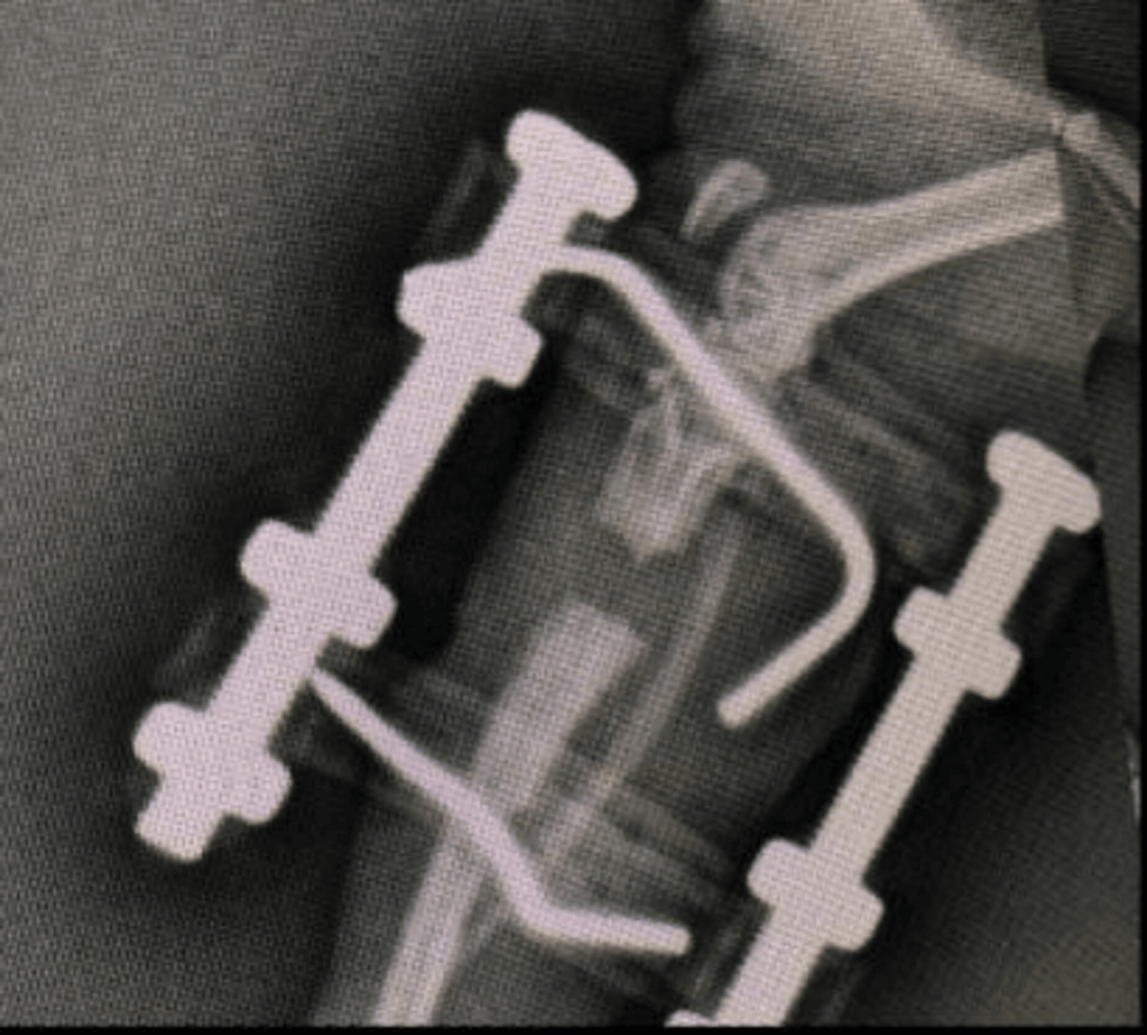
Post-Operative X-Ray Showing Lengthening in the Acute Distraction Rat Model

**Figure 6:**
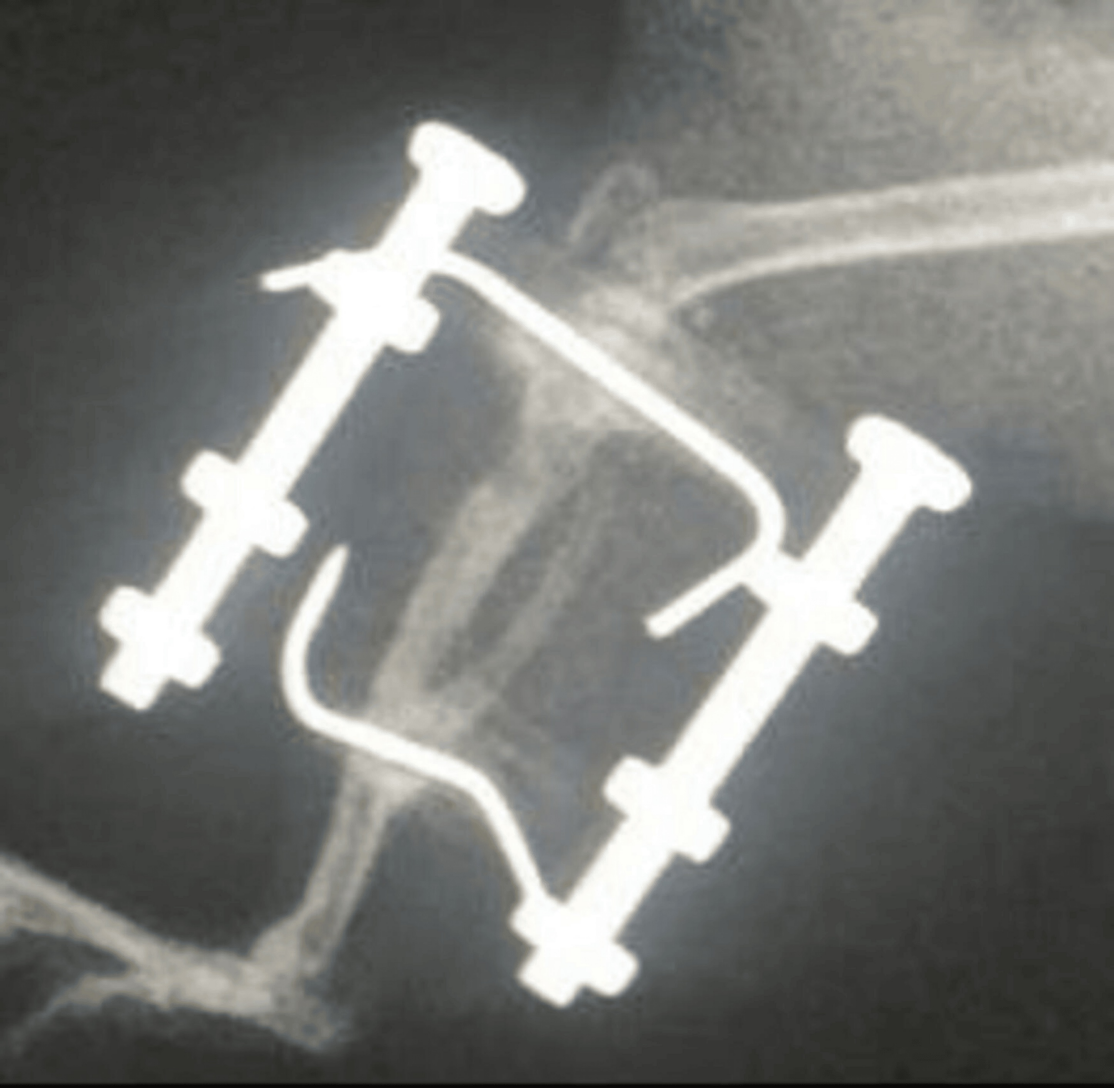
Bone Fill in the Distraction Area After Four Weeks of Waiting

To verify whether the system was functional, an acute distraction was tested on one rat model, and lengthening was successfully achieved (Figures 5-6).

Complications

**Figure 7:**
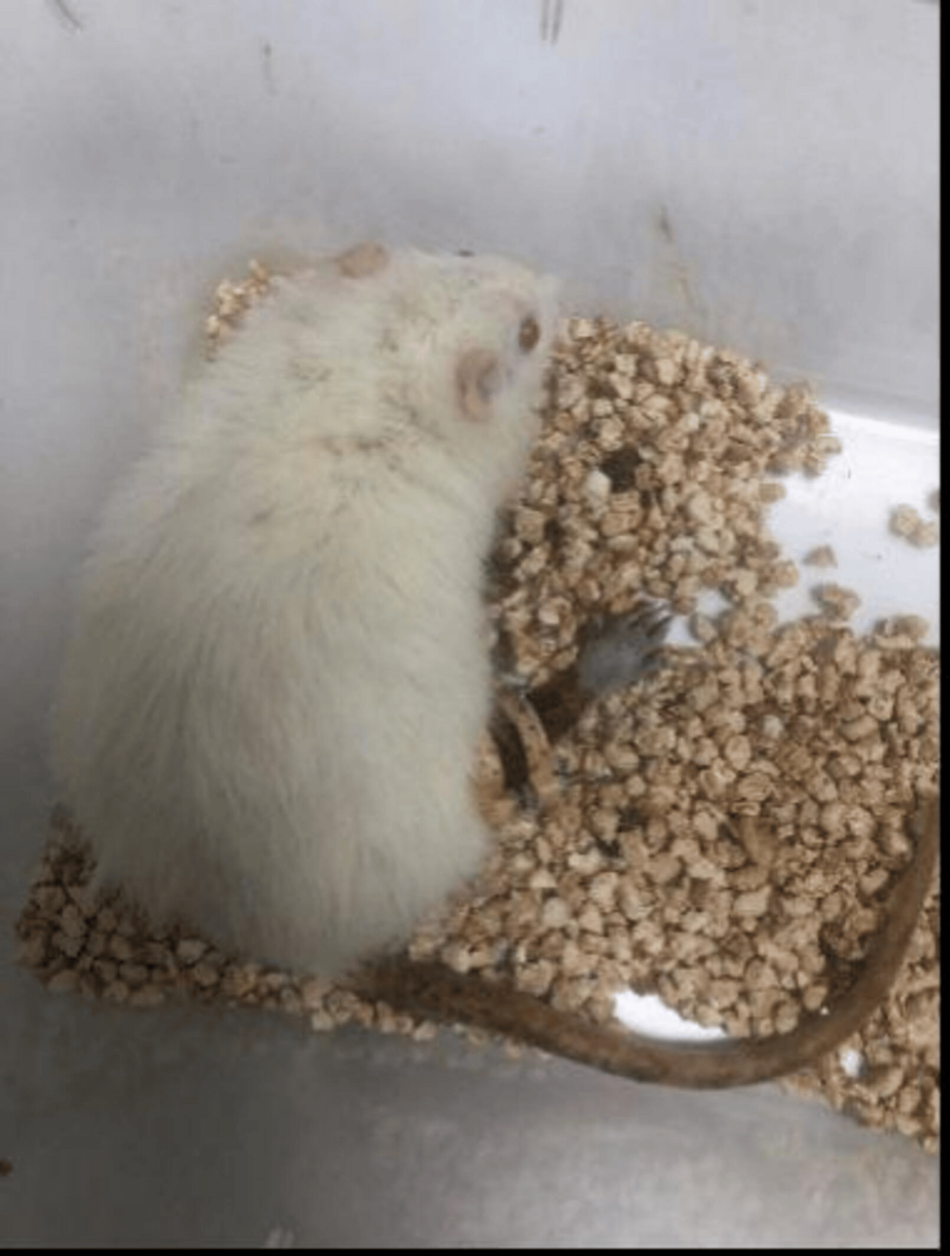
Foot Image of a Rat with Ecchymotic Appearance during Post Operative Care

In the case of one rat, circulation was compromised following the operation, and the extremity appeared ecchymotic. During follow-up, the extremity returned to its original condition (Figure 7).

**Figure 8:**
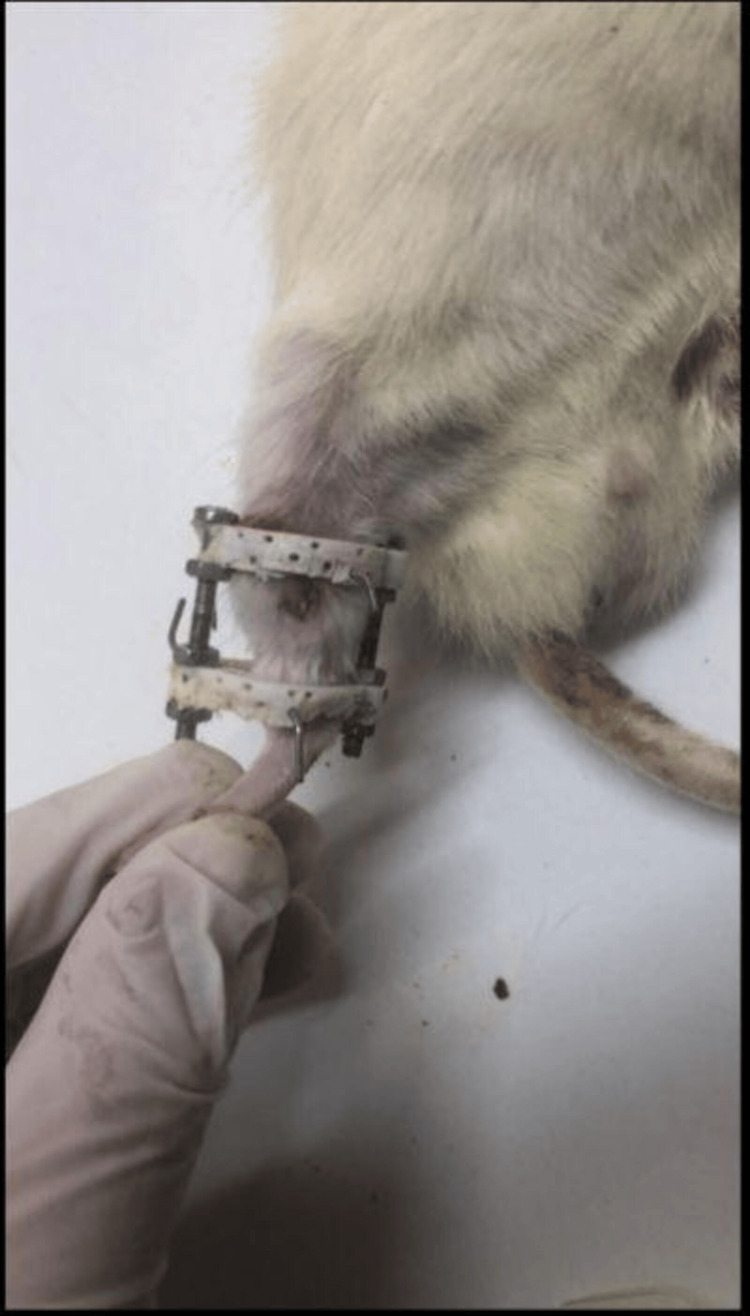
Soft Tissue Infection in a Rat Model

Superficial soft tissue infection developed and healed without any problems with daily dressing follow-ups. No postoperative antibiotic therapy was administered to any of the rat models. During follow-up, it was observed that the infections regressed with dressing management (Figure 8).

## Discussion

There are numerous studies on fracture healing, utilizing various animals. Some of the animals used in these studies include mice, rats, and rabbits [18]. In our study, rats were chosen due to their adequate number, ease of acquisition, rapid adaptation to external conditions, resistance to infections, and isogenic characteristics. The literature also indicates that similar studies are predominantly conducted using rats [19-25].

In various studies involving rat fracture models, several fixation methods have been used. One of these methods involves placing a retrograde K-wire through the knee region [18,26]. Implants that are introduced intramedullary without affecting knee and hip joint movements are commonly used. After creating a fracture, instead of using traditional retrograde K-wires, we utilized a circular Ilizarov method developed and produced by us. We believe that this method is more cost-effective and stable compared to alternatives.

Conditions that disrupt regional blood flow can negatively impact new bone formation. Therefore, techniques used should minimize local trauma, thermal necrosis, and disturbances in blood supply at the osteotomy site [27]. Successful distraction osteogenesis requires minimal trauma during osteotomy, good blood flow, stability of the fixation method, and an appropriate rate of rhythmic distraction [28]. In our experiment, the use of a circular fixator provides rigid fixation, thereby increasing the likelihood of successful bone healing.

Various osteotomy techniques have been described for distraction osteogenesis, including corticotomy, multiple drill osteotomy (De Bastiani technique), and Gigli wire osteotomy (Afghan technique) [27]. Ilizarov recommended the corticotomy technique because it promotes better callus formation [29]. Delloye et al. found no significant difference between corticotomy and other osteotomy techniques when comparing their effectiveness [8]. Kojimoto et al. (1988) observed that performing a subperiosteal osteotomy on the tibial diaphyses of 27 rabbits led to significant disruption in callus formation, indicating that the removal of the periosteum adversely affects bone healing. However, they noted that damage to the endosteum did not have a significant impact on callus formation. These findings suggest that preserving the periosteum is more crucial for successful bone lengthening than the method of corticotomy [30]. In our study, we also performed osteotomy while preserving the periosteum.

The metaphyseal region of the bone is considered an ideal site for osteotomy. This area provides better adaptation to distraction due to its blood supply and the density of spongy bone [31,32]. In his canine experiments on the placement of the Ilizarov osteotomy, he recommended performing the osteotomy in the metaphyseal region [33]. The diaphyseal region consists of more compact bone and less spongy bone compared to the metaphyseal region. In our study, osteotomies were performed in the metaphyseal regions of rat tibias.

Li et al. have reported that distraction frequency affects distraction osteogenesis in their studies. Animal studies were conducted with a distraction rate of 2 mm/day, which resulted in nonunion and delayed healing. If the distraction rate is between 0.3-0.5 mm/day, incomplete union occurs. In a study conducted in rabbits, a distraction rate of 1.3 mm/day was found to be too rapid, resulting in no bone formation. However, it has been determined that an optimal rate for rabbits is 0.7 mm/day, which is considered ideal for achieving effective bone formation [34].

In the extension model conducted on the femurs of Sprague-Dawley rats, a unilateral metal fixator system was used, with a distraction rate of 0.165 mm twice daily after a waiting period of seven days [35]. Aronson initiated distraction the day after osteotomy in rats, using a distraction rate of 0.2 mm twice daily [36]. We planned to perform distractions at a rate of 0.25 mm twice daily, following a seven-day waiting period.

Studies have indicated that pin-site infections are commonly encountered in distraction osteogenesis [37]. In our study, pin-site infections were observed in approximately three cases. During follow-up, it was noted that the infections resolved. Our complication rates are comparable to those of the literature. Even though our study was not affected by any serious complications, such as a severe deep tissue infection or osteomyelitis, it is advisable to perform more precise and meticulous surgery in order to reduce the risk of complications.

Mora and Forriove utilized a system in their distraction models that incorporated two half-rings connected to each other. In contrast, our setup features a circular design with the capability to insert K-wires from all sides [38]. In a study conducted by Isefuku et al. in 2000, a distraction model was established using mice. This model involved a fixator system applied to 12 adult male MF1 mice. Isefuku and colleagues utilized an improved half-ring system in their study. In our research, unlike the half-ring system designed by Isefuku et al., a perforated circular ring system was used, which facilitates fixation from all angles [15].

Utilizing a larger number of experimental animals in our study could be beneficial for monitoring postoperative outcomes. Additionally, performing biomechanical tests may provide more realistic results. We also recommend conducting angiography to assess vascular integrity and to evaluate any differences in vascularization following osteotomy.

The use of a novel, cost-effective device, successful distraction outcomes, and minimal complications are strengths of our study. Our study had a few limitations; namely, small sample size, absence of biomechanical testing, and the need for angiography to assess vascular changes.

## Conclusions

Current study, we investigated the extensions following osteotomies in the metaphyseal region of rat tibias on a weekly basis. Our observations included both macroscopic assessments and an analysis of potential complications. While we noted that some of the rat models developed complications such as infections and circulation problems during the process, it is important to highlight that none of these complications led to fatalities throughout the follow-up period. The lower incidence of severe complications, coupled with the enhanced radiological imaging capabilities provided by our extension models, demonstrated the robustness and reliability of our approach. This model, specifically targeting the metaphyseal region of rat tibias, proved to be effective and efficient, suggesting that it could be a valuable tool for future research applications. It shows significant potential for studies focusing on fracture healing, bone regeneration, or distraction osteogenesis. Furthermore, the relative simplicity and cost-effectiveness of our system compared to alternative models make it particularly appealing for use in experimental settings. By offering a reliable and affordable method for creating bone extension models, our approach could pave the way for broader adoption in preclinical studies aimed at advancing orthopedic and regenerative medicine.
